# Supplementary material for: An efficient moments-based inference method for within-host bacterial infection dynamics
Source: PLoS Comput Biol. 2017 Nov 20;13(11):e1005841. doi: 10.1371/journal.pcbi.1005841 (PMC5714343; doi:10.1371/journal.pcbi.1005841)
Supplement: S1 Supplementary Materials — (PDF) [file pcbi.1005841.s001.pdf]

# S1 Supplementary Materials

## S1.1 Matrix Exponential Evaluation of Integral

Define the matrix $K$ to be,

$$K = \begin{pmatrix} C & B \\ 0 & A \end{pmatrix},$$

where matrices $C$, $B$ and $A$ are as before. As all powers of $K$ have the same block-triangular structure, we can denote $\exp(tK)$ as,

$$\exp(tK) = \begin{pmatrix} F(t) & H(t) \\ 0 & G(t) \end{pmatrix}.$$

Taking the derivative of $\exp(tK)$ with respect to $t$ gives,

$$\frac{d}{dt}\exp(tK) = K\exp(tK),$$

and hence expressions for each of $F(t), G(t)$, and $H(t)$,

$$\begin{aligned} \frac{dF(t)}{dt} =& CF(t), \\ \frac{dG(t)}{dt} =& AG(t), \text{ and} \\ \frac{dH(t)}{dt} =& CH(t) + BG(t). \end{aligned}$$

Solving for $H(t)$ gives $\exp(Ct) \times \left[\int_0^t \exp(-Cs) \times B \times \exp(As) ds\right]$, which we can use to evaluate the second moments of the stochastic system exactly. Thus, for a given set of model parameters, we can solve the first two moments exactly.

## S1.2 Derivation of Hellinger and Kullback-Leibler Divergences for Multivariate Normal Distributions

For distributions $P$ and $Q$ of a continuous random variable, the Kullback-Leibler divergence [1, 2] is defined as

$$\Delta_K(P||Q) = \int_{\mathbb{R}^d} p(\mathbf{x}) \log \frac{p(\mathbf{x})}{q(\mathbf{x})} d\mathbf{x}\ .$$

The Rényi divergence of order $r$ [3] is given by

$$D_r(P||Q) = \frac{1}{r-1} \log \int_{\mathbb{R}^d} p(\mathbf{x})^r q(\mathbf{x})^{1-r} d\mathbf{x} \ ,$$

and this equals the Kullback-Leibler divergence in the limit $r \to 1$ [4]:

$$\lim_{r \to 1} D_r(P||Q) = \Delta_K(P||Q) \ . \tag{1}$$

Another measure of divergence between $P$ and $Q$ is the squared Hellinger distance [5] defined as

$$\Delta_H^2(P||Q) = \frac{1}{2} \int_{\mathbb{R}^d} \left( \sqrt{p(\mathbf{x})} - \sqrt{q(\mathbf{x})} \right)^2 d\mathbf{x} \ . \tag{2}$$

We derive expressions for $\Delta_K$ and $\Delta_H^2$ with respect to $P$ and $Q$ as multivariate normal distributions. In both cases, the following lemma will be used in the proofs.

**Lemma 1:**

*If $P$ and $Q$ are multivariate normal distributions with parameters $(\boldsymbol{\mu}_1, \boldsymbol{\Sigma}_1)$ and $(\boldsymbol{\mu}_2, \boldsymbol{\Sigma}_2)$, respectively, then*

$$\int_{\mathbb{R}^d} p(\mathbf{x})^r q(\mathbf{x})^{1-r} d\mathbf{x} = \frac{|r\boldsymbol{\Sigma}_2 + (1-r)\boldsymbol{\Sigma}_1|^{-1/2}}{|\boldsymbol{\Sigma}_1|^{(r-1)/2} |\boldsymbol{\Sigma}_2|^{-r/2}} \times \exp\left\{ \frac{r(r-1)}{2} \boldsymbol{\mu}^T \left( r\boldsymbol{\Sigma}_2 + (1-r)\boldsymbol{\Sigma}_1 \right)^{-1} \boldsymbol{\mu} \right\}$$

*[6, pp.45-46].*

**Theorem 1:**

*If $P$ and $Q$ are d-dimensional multivariate normal distributions with parameters $(\boldsymbol{\mu}_1, \boldsymbol{\Sigma}_1)$ and $(\boldsymbol{\mu}_2, \boldsymbol{\Sigma}_2)$, respectively, then*

$$\Delta_K(P||Q) = \frac{1}{2} \left[ \mathrm{tr}\left( \boldsymbol{\Sigma}_2^{-1} \boldsymbol{\Sigma}_1 \right) - d + \boldsymbol{\mu}^T \boldsymbol{\Sigma}_2^{-1} \boldsymbol{\mu} + \log \frac{|\boldsymbol{\Sigma}_2|}{|\boldsymbol{\Sigma}_1|} \right] \ .$$

Proof From the limit expression of equation (1), we have

$$\begin{aligned} \Delta_K(P||Q) &= \lim_{r \to 1} D_r(P||Q) \\ &= \lim_{r \to 1} \frac{1}{r-1} \log \int_{\mathbb{R}^d} p(\mathbf{x})^r q(\mathbf{x})^{1-r} d\mathbf{x} \ , \end{aligned}$$

therefore, from Lemma 1,

$$\Delta_K(P||Q) = \lim_{r\to 1} \frac{r}{2}\, \boldsymbol{\mu}^T \left(r\boldsymbol{\Sigma}_2 + (1-r)\boldsymbol{\Sigma}_1\right)^{-1} \boldsymbol{\mu}$$
$$-\frac{1}{2} \lim_{r\to 1} \frac{1}{r-1} \log\left\{\frac{|r\boldsymbol{\Sigma}_2 + (1-r)\boldsymbol{\Sigma}_1|}{|\boldsymbol{\Sigma}_1|^{1-r}|\boldsymbol{\Sigma}_2|^r}\right\}$$

to give

$$\Delta_K(P||Q) = \frac{1}{2}\, \boldsymbol{\mu}^T \left(\boldsymbol{\Sigma}_2\right)^{-1} \boldsymbol{\mu} - \frac{1}{2} \lim_{r\to 1} \frac{1}{r-1} \log\left\{\frac{|r\boldsymbol{\Sigma}_2 + (1-r)\boldsymbol{\Sigma}_1|}{|\boldsymbol{\Sigma}_1|^{1-r}|\boldsymbol{\Sigma}_2|^r}\right\} . \tag{3}$$

We can solve the limit within equation (3) as follows. Let

$$a(r) = \log\left\{\frac{|r\boldsymbol{\Sigma}_2 + (1-r)\boldsymbol{\Sigma}_1|}{|\boldsymbol{\Sigma}_1|^{1-r}|\boldsymbol{\Sigma}_2|^r}\right\}$$

and consider $\partial a(r)/\partial r$. This partial derivative can be solved through the use of the fact that

$$\frac{\partial|\mathbf{A}|}{\partial x} = |\mathbf{A}|\mathrm{tr}\left(\mathbf{A}^{-1}\frac{\partial \mathbf{A}}{\partial x}\right)$$

for any invertible matrix $\mathbf{A}$; consequently,

$$\frac{\partial a(r)}{\partial r} = \mathrm{tr}\big(\left(r\boldsymbol{\Sigma}_2 + (1-r)\boldsymbol{\Sigma}_1\right)^{-1}\left(\boldsymbol{\Sigma}_2 - \boldsymbol{\Sigma}_1\right)\big) - \log\frac{|\boldsymbol{\Sigma}_2|}{|\boldsymbol{\Sigma}_1|} .$$

Given that $\lim_{r\to 1} a(r) = \log 1 = 0$, $\lim_{r\to 1} r - 1 = 0$ and $\partial a(r)/\partial r$ exists, we can determine the limit within equation (3) via l'Hôpital's rule:

$$\begin{aligned}
\lim_{r\to 1} \frac{a(r)}{r-1} &= \lim_{r\to 1} \frac{\partial a(r)/\partial r}{\partial(r-1)/\partial r} \\
&= \lim_{r\to 1} \frac{\partial a(r)}{\partial r} \\
&= \mathrm{tr}\big(\boldsymbol{\Sigma}_2^{-1}\left(\boldsymbol{\Sigma}_2 - \boldsymbol{\Sigma}_1\right)\big) - \log\frac{|\boldsymbol{\Sigma}_2|}{|\boldsymbol{\Sigma}_1|} \\
&= -\mathrm{tr}\big(\boldsymbol{\Sigma}_2^{-1}\boldsymbol{\Sigma}_1 - \mathbf{I}_d\big) - \log\frac{|\boldsymbol{\Sigma}_2|}{|\boldsymbol{\Sigma}_1|} \\
&= -\mathrm{tr}\big(\boldsymbol{\Sigma}_2^{-1}\boldsymbol{\Sigma}_1\big) + d - \log\frac{|\boldsymbol{\Sigma}_2|}{|\boldsymbol{\Sigma}_1|} ,
\end{aligned} \tag{4}$$

therefore, upon substituting (4) into (3), we have

$$\Delta_K(P||Q) = \frac{1}{2}\left[\mathrm{tr}\big(\boldsymbol{\Sigma}_2^{-1}\boldsymbol{\Sigma}_1\big) - d + \boldsymbol{\mu}^T\boldsymbol{\Sigma}_2^{-1}\boldsymbol{\mu} + \log\frac{|\boldsymbol{\Sigma}_2|}{|\boldsymbol{\Sigma}_1|}\right] . \qquad \blacksquare$$

**Theorem 2:**

*If $P$ and $Q$ are multivariate normal distributions with parameters $(\boldsymbol{\mu}_1, \boldsymbol{\Sigma}_1)$ and $(\boldsymbol{\mu}_2, \boldsymbol{\Sigma}_2)$, respectively, then*

$$\Delta_H^2(P||Q) = 1 - \exp\left\{-\frac{1}{8}\boldsymbol{\mu}^T\left(\frac{\boldsymbol{\Sigma}_2+\boldsymbol{\Sigma}_1}{2}\right)^{-1}\boldsymbol{\mu}\right\} \times \left(\frac{|\frac{\boldsymbol{\Sigma}_2+\boldsymbol{\Sigma}_1}{2}|}{\sqrt{|\boldsymbol{\Sigma}_1||\boldsymbol{\Sigma}_2|}}\right)^{-1/2} .$$

Proof We start with the definition of $\Delta_H^2$ given by equation (2):

$$\begin{aligned}\Delta_H^2(P||Q) &= \frac{1}{2}\int_{\mathbb{R}^d}\left(\sqrt{p(\mathbf{x})}-\sqrt{q(\mathbf{x})}\right)^2 d\mathbf{x}\\ &= \frac{1}{2}\left(2-2\int_{\mathbb{R}^d}\sqrt{p(\mathbf{x})}\sqrt{q(\mathbf{x})}d\mathbf{x}\right)\\ &= 1-\int_{\mathbb{R}^d}\sqrt{p(\mathbf{x})}\sqrt{q(\mathbf{x})}d\mathbf{x}\ .\end{aligned}$$

But, from Lemma 1,

$$\begin{aligned}\int_{\mathbb{R}^d} p(\mathbf{x})^{1/2}q(\mathbf{x})^{1/2}d\mathbf{x} = &\frac{|\frac{1}{2}\boldsymbol{\Sigma}_2+\frac{1}{2}\boldsymbol{\Sigma}_1|^{-1/2}}{|\boldsymbol{\Sigma}_1|^{-1/4}|\boldsymbol{\Sigma}_2|^{-1/4}} \times\\ &\exp\left\{-\frac{1}{8}\boldsymbol{\mu}^T\left(\frac{1}{2}\boldsymbol{\Sigma}_2+\frac{1}{2}\boldsymbol{\Sigma}_1\right)^{-1}\boldsymbol{\mu}\right\} ,\end{aligned}$$

therefore,

$$\Delta_H^2(P||Q) = 1 - \exp\left\{-\frac{1}{8}\boldsymbol{\mu}^T\left(\frac{\boldsymbol{\Sigma}_2+\boldsymbol{\Sigma}_1}{2}\right)^{-1}\boldsymbol{\mu}\right\} \times \left(\frac{\left|\frac{\boldsymbol{\Sigma}_2+\boldsymbol{\Sigma}_1}{2}\right|}{\sqrt{|\boldsymbol{\Sigma}_1||\boldsymbol{\Sigma}_2|}}\right)^{-1/2} . \quad \blacksquare$$

## S1.3 Moment computation for radial and linear networks

Figures S1 and S2 show the calculation of the moments and computation time, respectively, using either Gillespie simulations or the moments equation, for a range of different size experiments (i.e., from 10 to 1000 simulations per experiment).

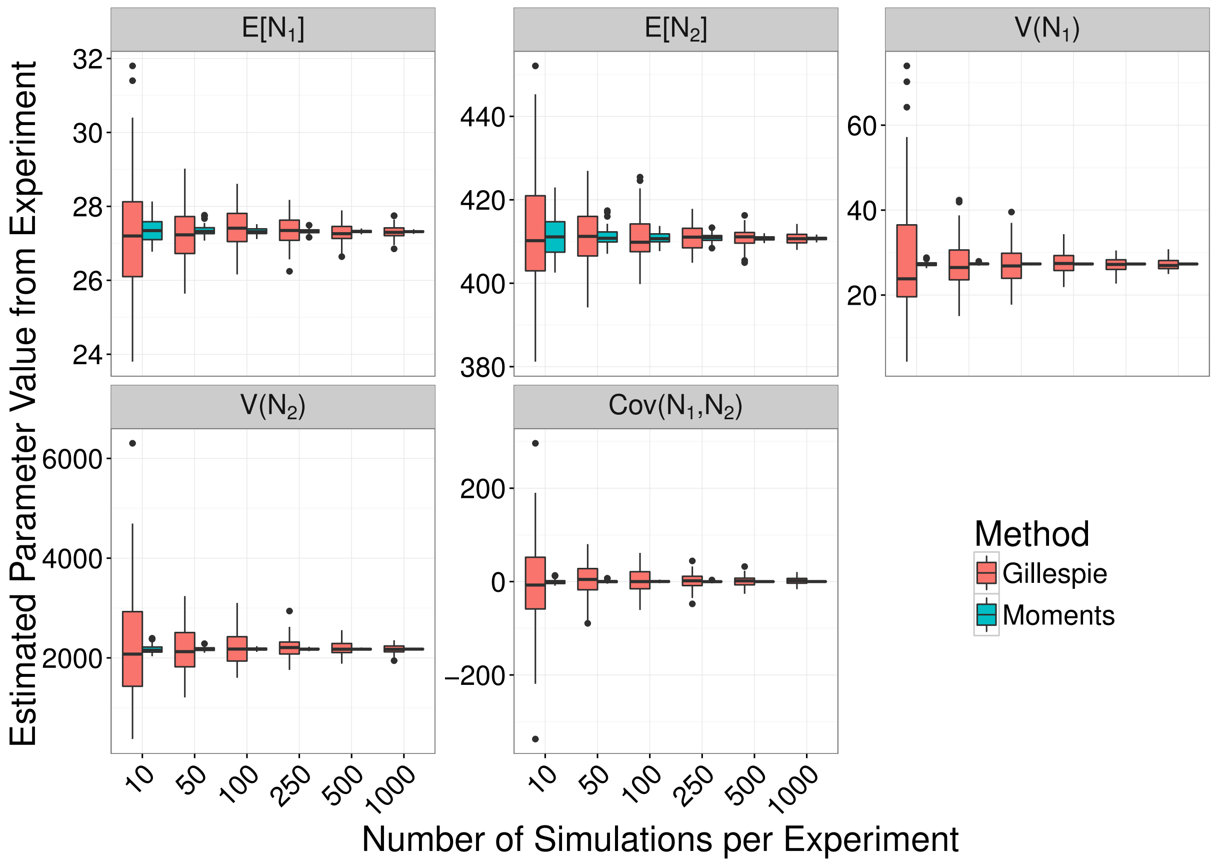


**Figure S1:** Box plots of estimated moments from 100 simulated experiments using the two-compartment birth-death-migration model (Figure 3a). For each experiment (of size 10, 50, 100, 250, 500 or 1000), the lower moments of variables $N_1$ and $N_2$ at time $t = 6$ were calculated from Gillespie simulations (red boxes) or from the moments equations ("direct method", blue boxes), starting from Poisson-distributed initial conditions. The stated number of simulations per experiments is equivalent to the total number of replicate observation ($A_i T$ at time point $\tau_i$ as per section 2.1).

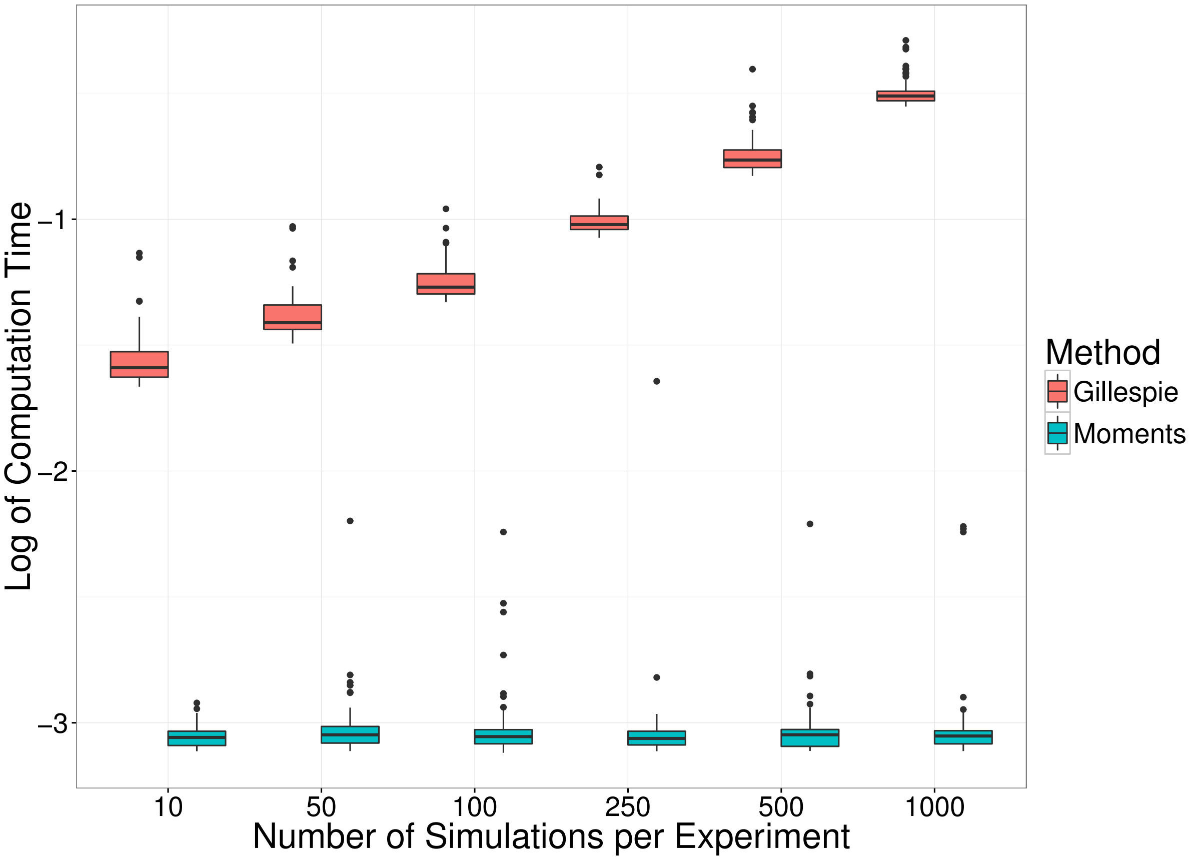


**Figure S2:** Box plots of computation time to evaluate the moments from 100 simulated experiments of the two-compartment birth-death-migration model, as per Figure S1. The vertical axis shows the logarithm in base 10 of running times in seconds.

Figures S3 and S4 show the calculation of the moments (and computation time) using either Gillespie simulations or the moments equation, for a range of different size experiments (i.e., from 10 to 1000 simulations per experiment).

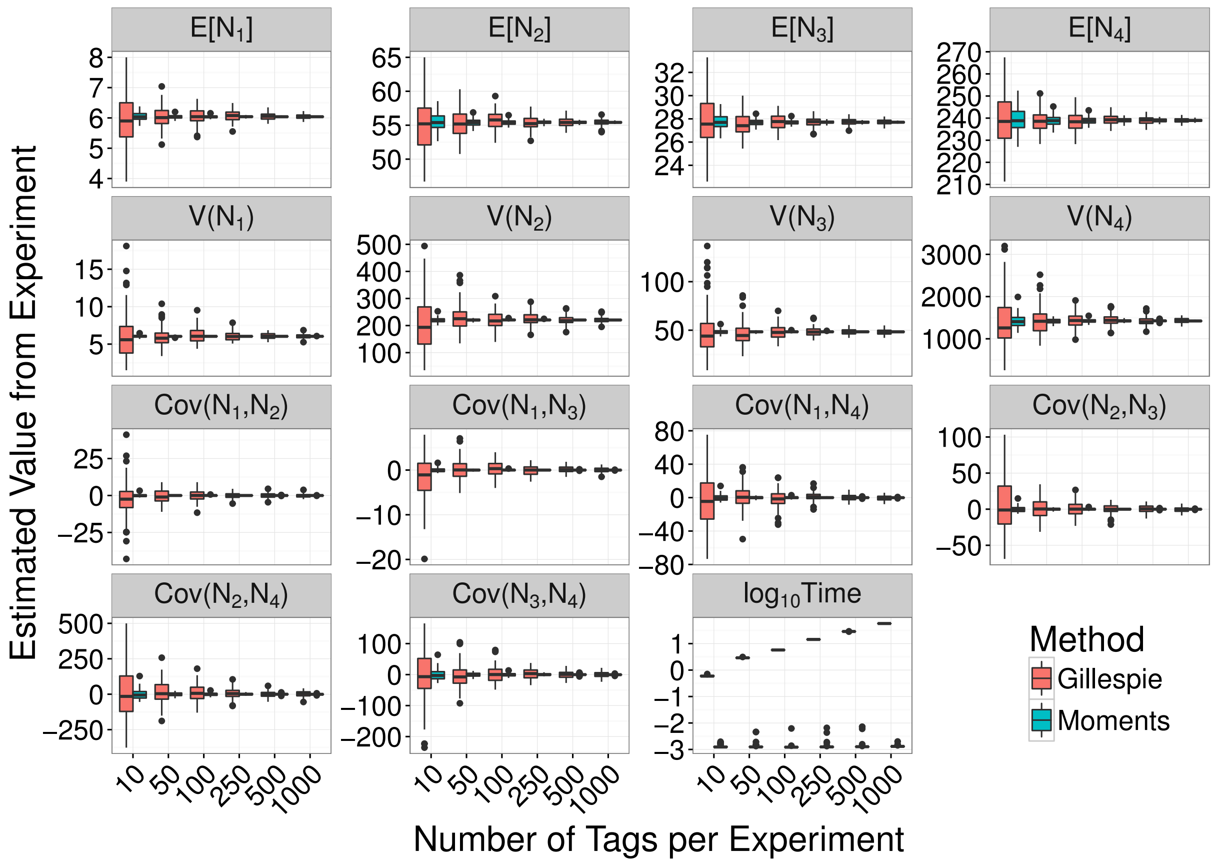


**Figure S3:** Box plots of estimated moments and log computation time from 100 simulated experiments of the radial network with four compartments, each of size 10, 50, 100, 250, 500 or 1000, calculated from the simulations and from the moments equations using the initial conditions.

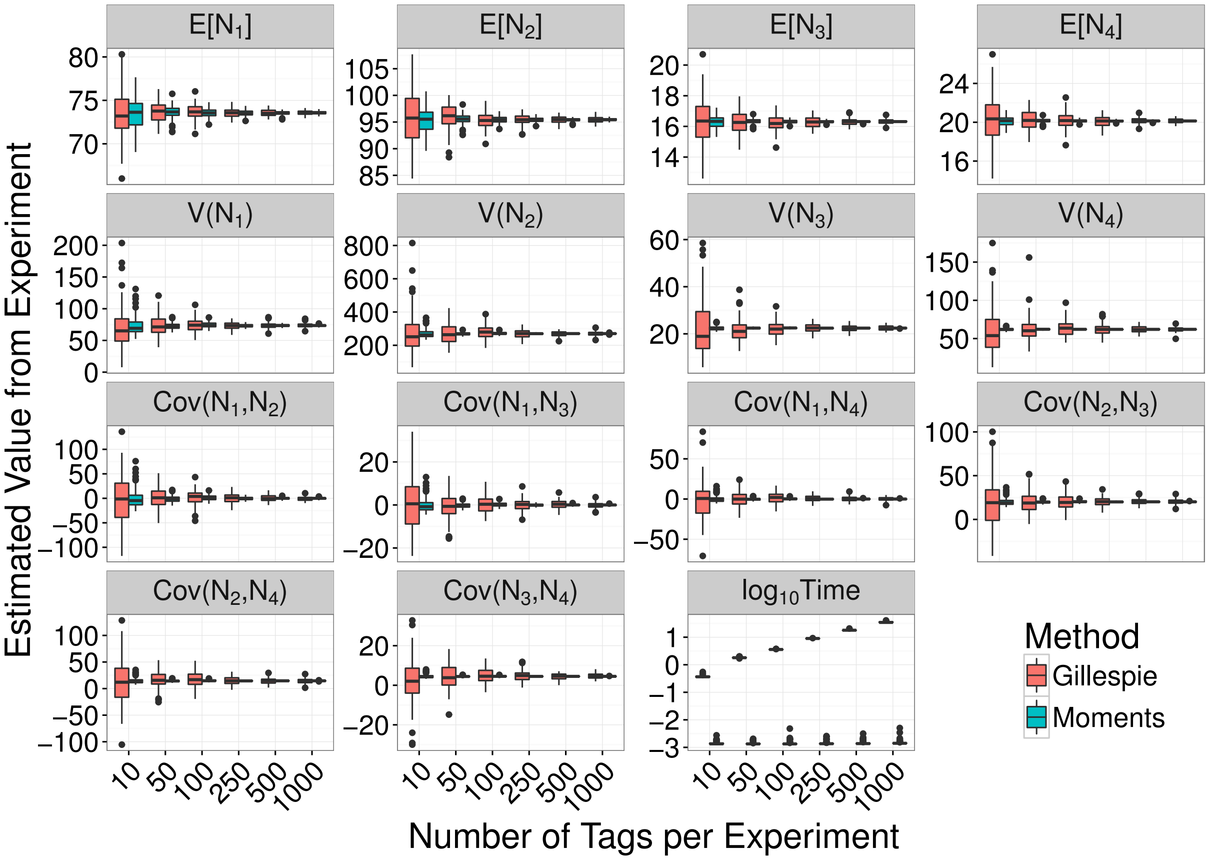


**Figure S4:** Box plots of estimated moments from 100 simulated experiments of the linear network with four compartments, each of size 10, 50, 100, 250, 500 or 1000, calculated from the simulations and from the moments equations using the initial conditions.

## S1.4 Comparison of Divergence Measure by Scenario

Figures S5, S6 and S7 show the averaged mean absolute relative error across each scenario for the four different measures. The horizontal lines represent the mean for each measure, and the error bars show ± one standard error (across the 100 simulations, at each observation time).

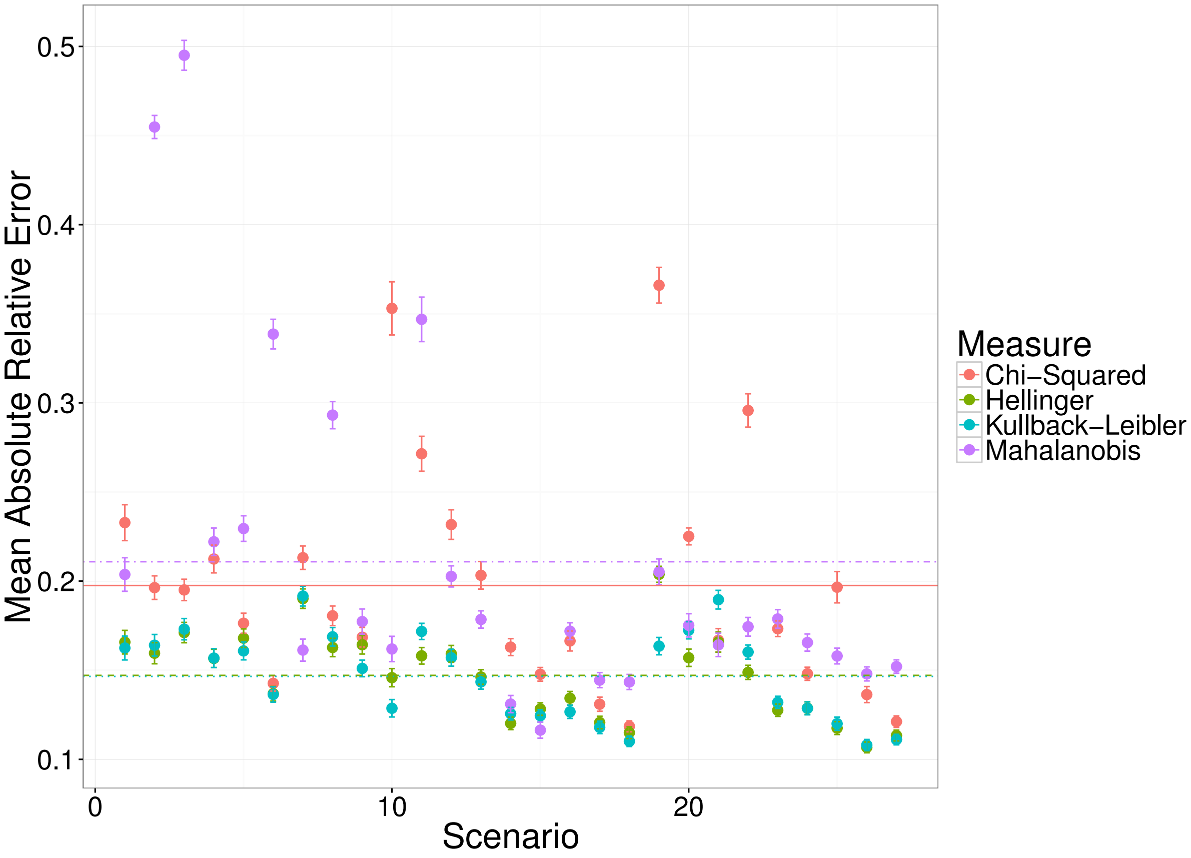


**Figure S5:** Mean Absolute Relative Error of parameter estimates of the simple model, for each divergence measure across each scenario, averaged across each of the 100 simulations at each observation time. The horizontal lines correspond to the average mean absolute relative error for each method.

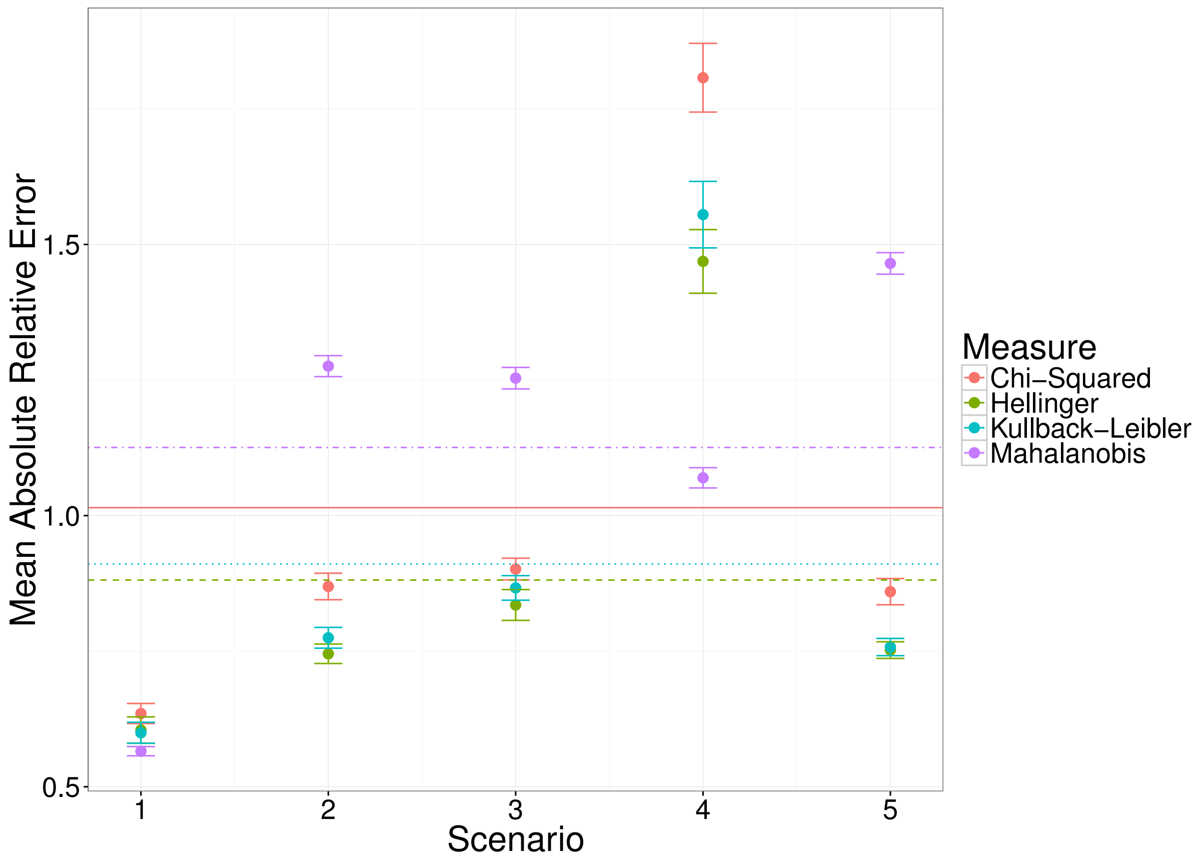


**Figure S6:** Mean Absolute Relative Error of parameter estimates of the linear model, for each divergence measure across each scenario, averaged across each of the 100 simulations at each observation time. The horizontal lines correspond to the average mean absolute relative error for each method.

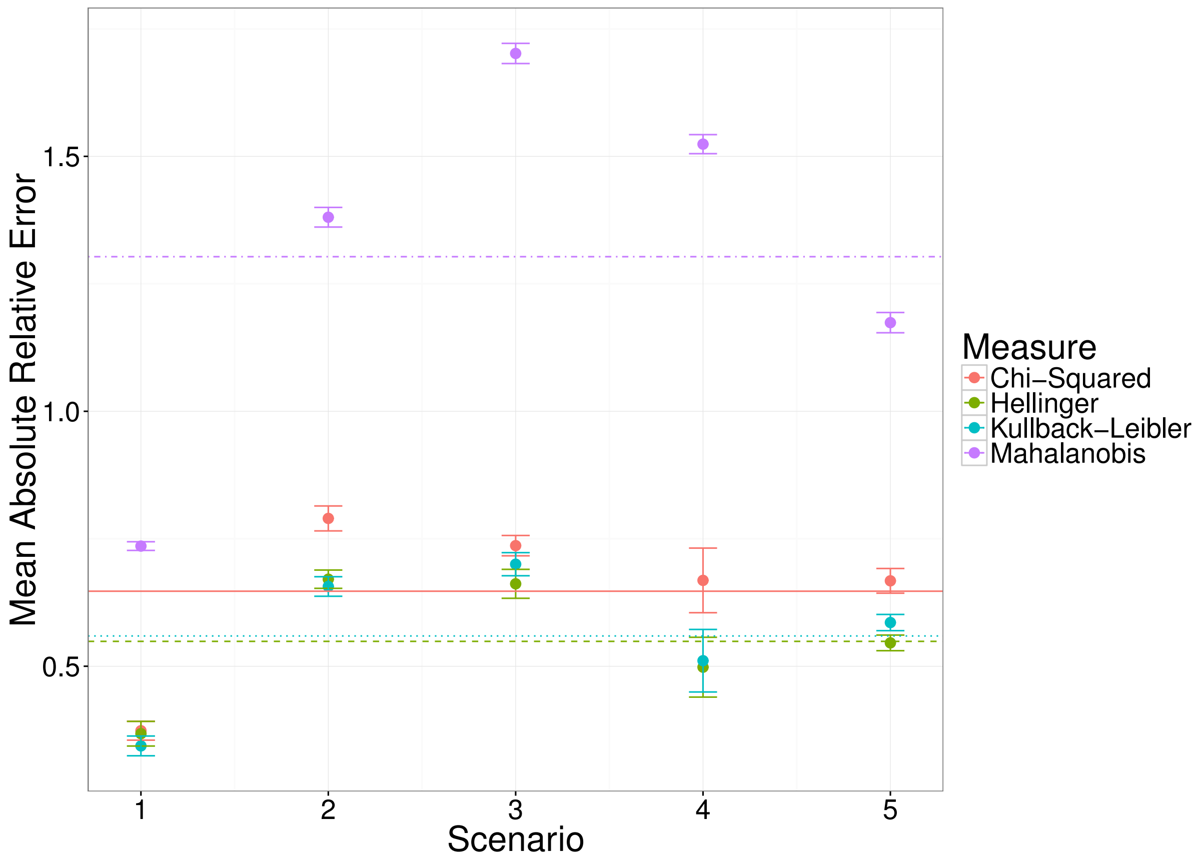


**Figure S7:** Mean Absolute Relative Error of parameter estimates of the radial model, for each divergence measure across each scenario, averaged across each of the 100 simulations at each observation time. The horizontal lines correspond to the average mean absolute relative error for each method.

## S1.5 Parameter estimates for model with two-way migration

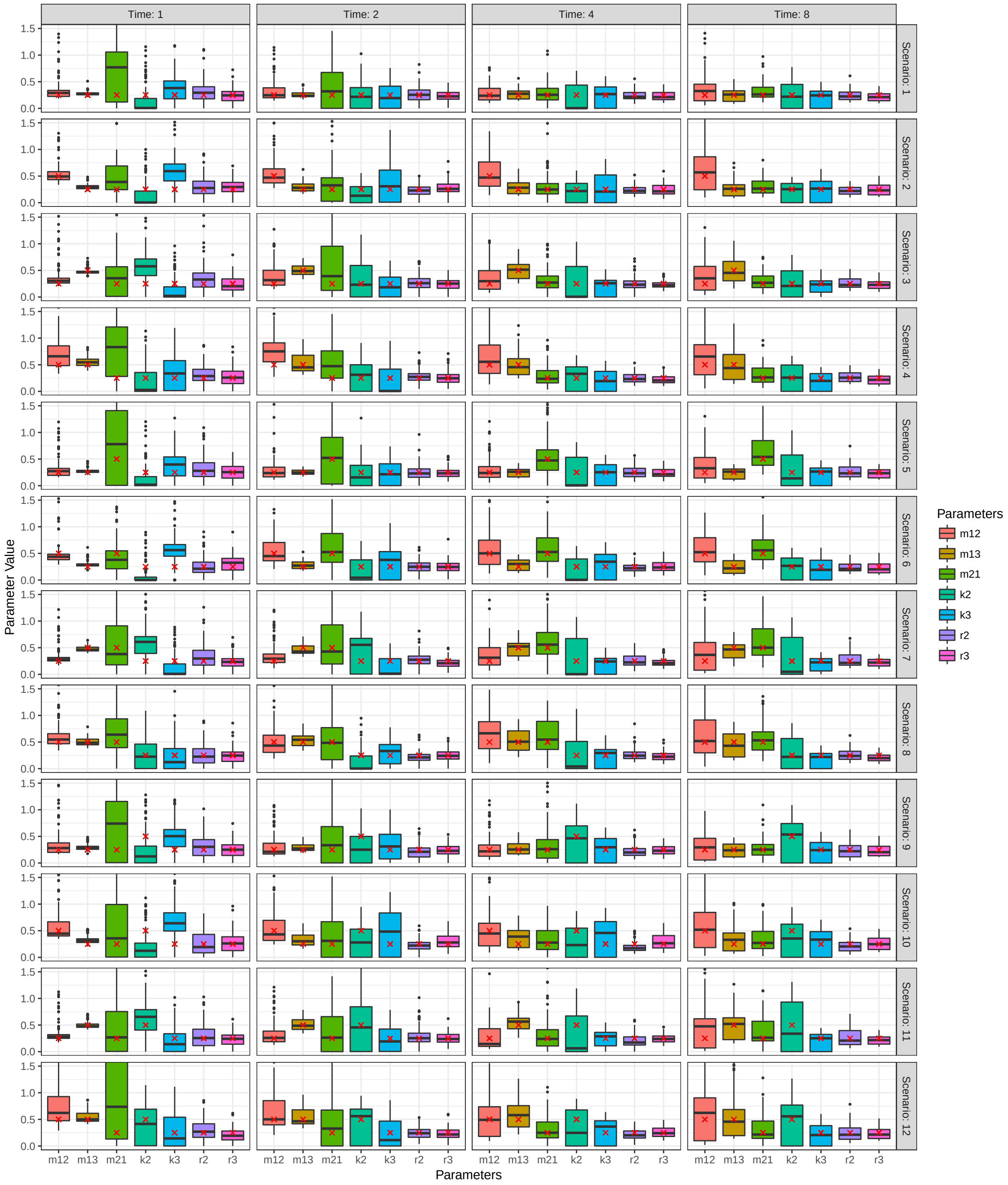


**Figure S8:** Distribution of estimates for the seven parameters of the two-way-migration model described in Section 3.3. The boxplots in each panel shows the results of MDE applied to 100 datasets consisting of 100 simulations each. Panels are ordered by observation time (columns) and parameter values (12 "scenarios" along the rows). The target parameter values for each scenario are shown by the red crosses.

## S1.6 Multiple Observations

It is often of interest to estimate parameters of a model combining information obtained at multiple observation times. Here, we test minimising a different function of the divergences arising from the different observation times. In particular, we consider the sum of divergences at both times, the product of the divergences, the maximum of the two divergences, and each of the two divergences individually.

As with the comparison of the four divergence measures in Section 3.2, we consider a range of parameter values, and assess how well each function of the Kullback-Leibler divergence performs inference with regards to the mean absolute relative error in the estimated parameter values. Each simulation within each scenario is made up of 100 replicates (e.g., WITS). The initial conditions for the first organ are randomly generated from a Poisson distribution with rate parameter 200, and the other organ is set to zero.

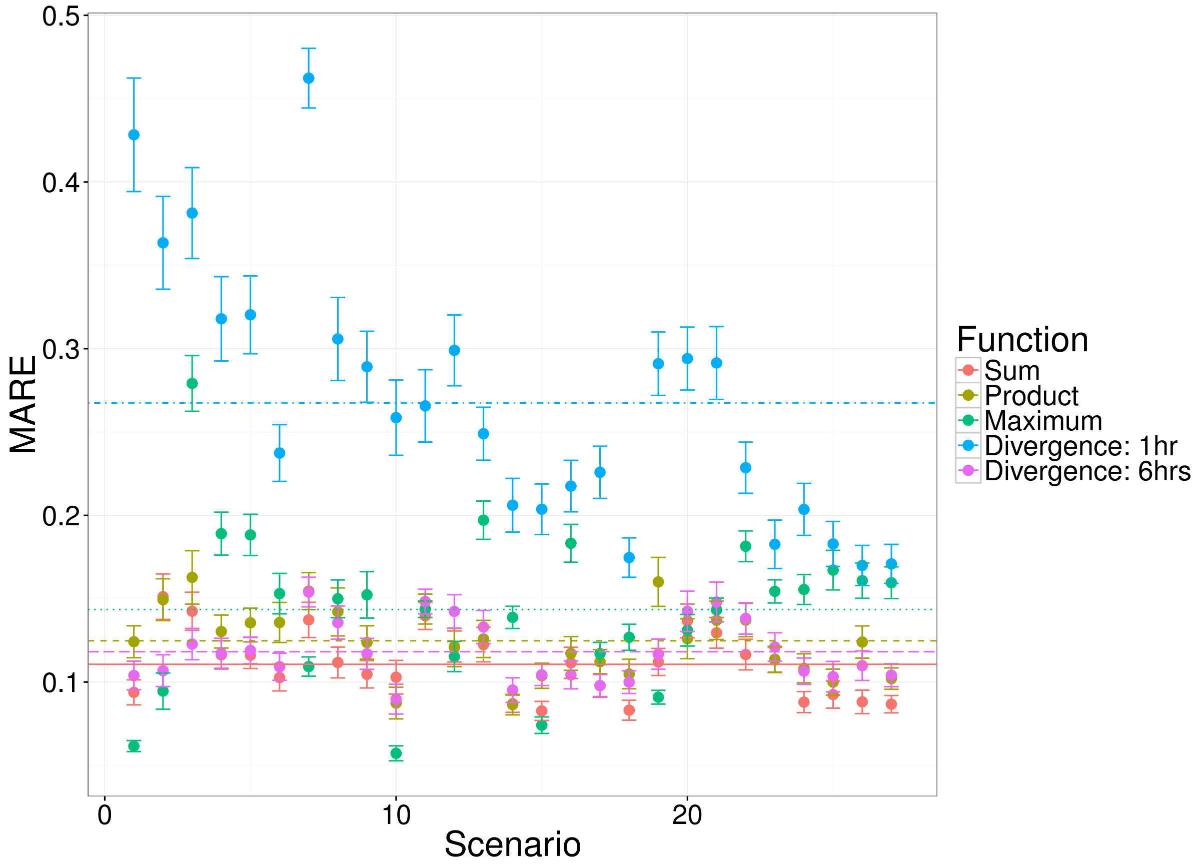


**Figure S9:** Mean Absolute Relative Error of parameter estimates for each divergence composition function across each scenario. The error bars represent ± one standard error across the 100 simulations under each scenario. The horizontal lines correspond to the overall mean for each composition function.

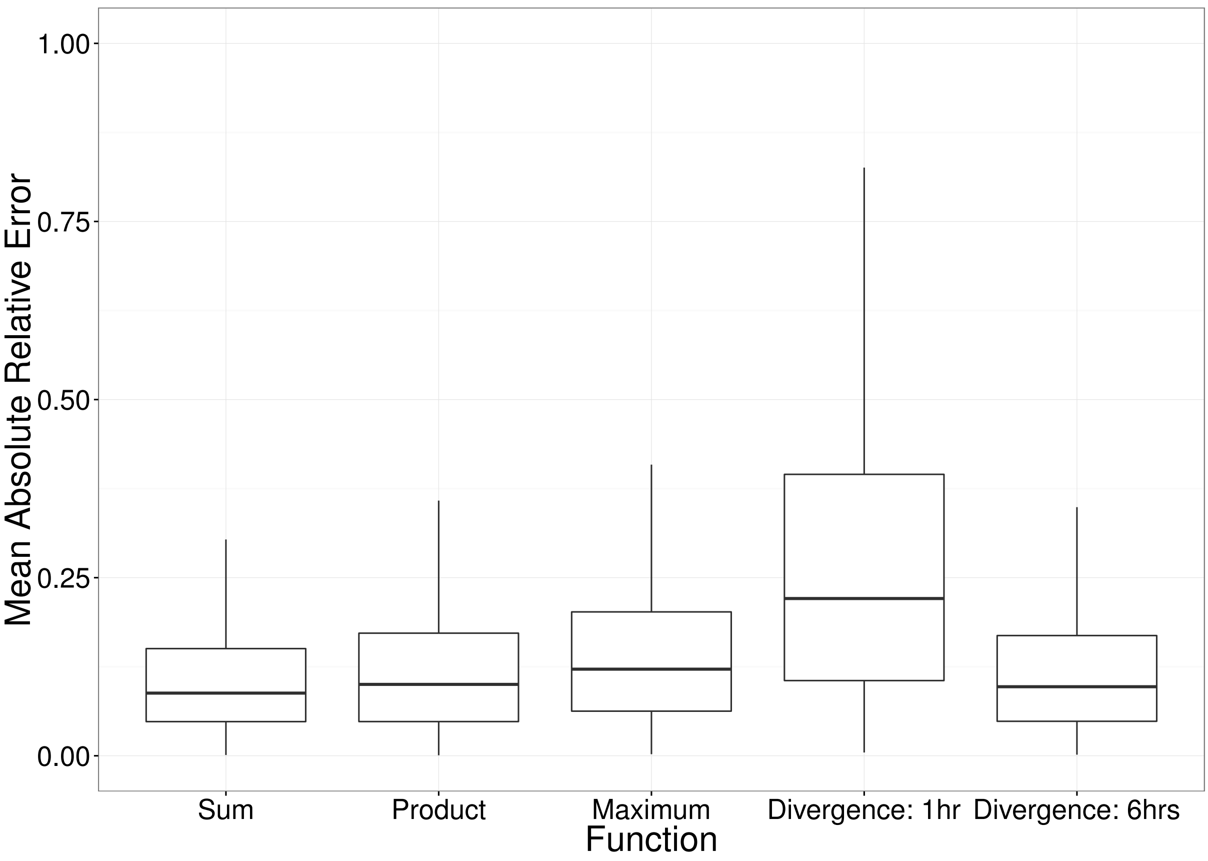


**Figure S10:** Box plots of the Mean Absolute Relative Error of parameter estimates for each divergence summary.

Figures S9 and S10 demonstrate that the sum of the divergences corresponds to the smallest mean absolute relative error in the parameter estimation, on average.

Note that the Kullback-Leibler divergence could be estimated in the other direction – that is, from the observed data to the model, rather than from the model to the data as we have considered thus far. We have considered the MDE tool with this alternate parameterisation and the results were very similar, and thus, are omitted.

## S1.7 Inference: Simulation Study

Parameter estimate results for all scenarios, across all observation times.

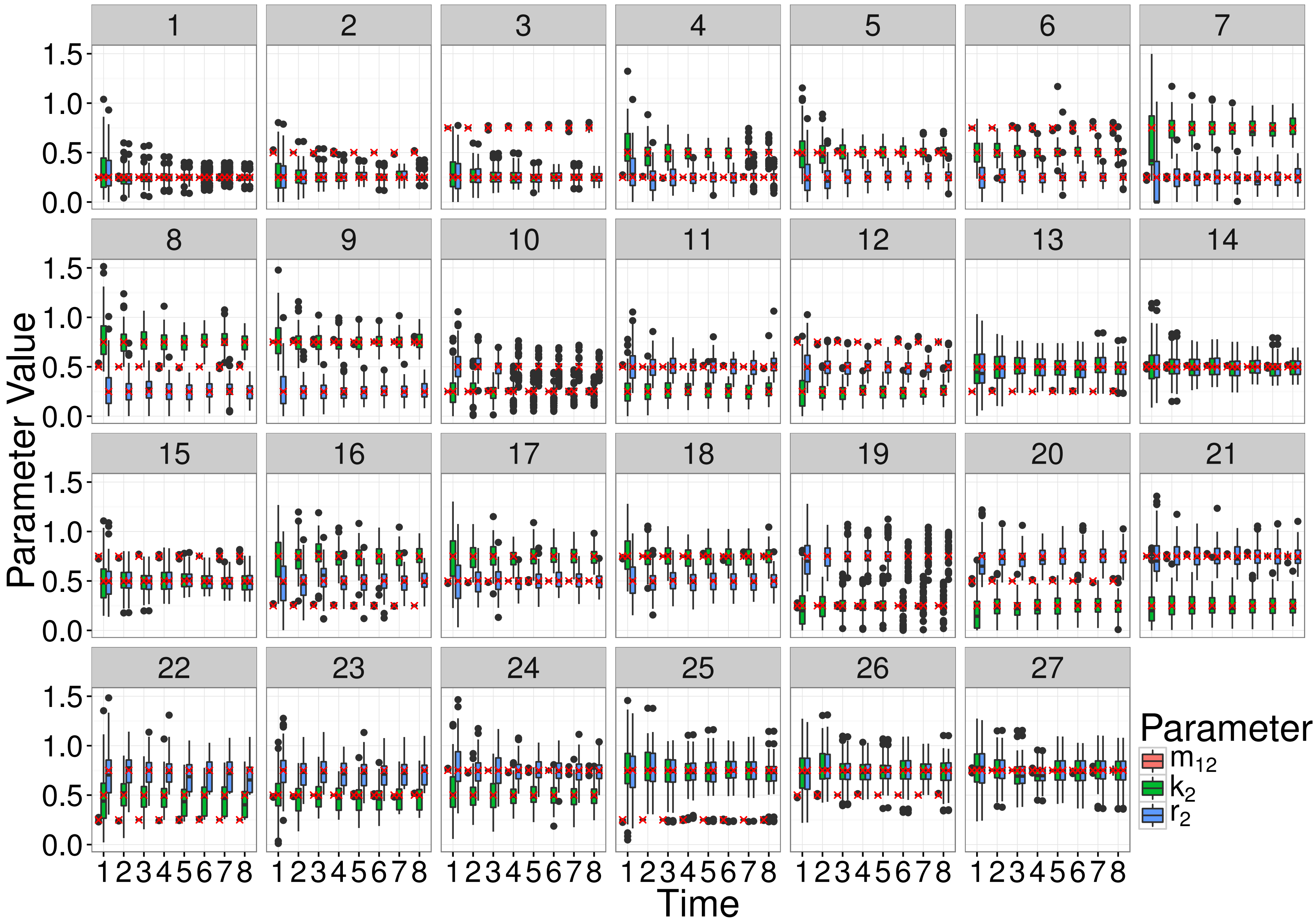


**Figure S11:** Box plots of MDE estimates corresponding to simulated data at $\tau_1 = 2$, where the true parameter is denoted by the red cross.

## S1.8 Moments of Blood, Liver, Spleen System

The first two moments ($M_1$ and $M_2$, respectively), of the blood, liver, spleen system follow a closed system of nine differential equations. With respect to the liver and spleen, denote the rate at which bacteria: move from the blood (clearance) as $c_L$ and $c_S$; move back into the blood (emmigration) as $e_L$ and $e_S$; replicate as $r_L$ and $r_S$; and, are killed as $k_L$ and $k_S$.

We can write the differential equations corresponding to the first moment in matrix form as the following:

$$\frac{\partial M_1(t)}{\partial t} = A \times M_1(t), \tag{5}$$

where,

$$M_1(t) = \begin{pmatrix} E\left[N_B(t)\right] \\ E\left[N_L(t)\right] \\ E\left[N_S(t)\right] \end{pmatrix} \quad \text{and} \quad A = \begin{pmatrix} -(c_L + c_S) & e_L & e_S \\ c_L & r_L - k_L - e_L & 0 \\ c_S & 0 & r_S - k_S - e_S \end{pmatrix}. \tag{6}$$

Similarly, we can write the second moments as:

$$\frac{\partial M_2(t)}{\partial t} = C \times M_2(t) + B \times \exp(tA) \times M_1(0),$$

where $M_1(0)$ are the initial moments of the system,

$$B = \begin{pmatrix} c_L + c_S & e_L & e_S \\ c_L & r_L + k_L + e_L & 0 \\ c_S & 0 & r_S + k_S + e_S \\ -c_L & -e_L & 0 \\ -c_S & 0 & -e_S \\ 0 & 0 & 0 \end{pmatrix},$$

and,

$$C = \begin{pmatrix} -2\kappa & 0 & 0 & 2e_L & 2e_S & 0 \\ 0 & 2\Delta_L & 0 & 2c_L & 0 & 0 \\ 0 & 0 & 2\Delta_S & 0 & 2c_S & 0 \\ c_L & e_L & 0 & \Delta_L - \kappa & 0 & e_S \\ c_S & 0 & e_S & 0 & \Delta_S - \kappa & e_L \\ 0 & 0 & 0 & c_L & c_S & \Delta_L + \Delta_S \end{pmatrix}$$

with $\Delta_L = r_L - k_L - e_L$, $\Delta_S = r_S - k_S - e_S$, and $\kappa = c_L + c_S$. The solutions to these equations are given by equations (3) and (4) in the main text.

# References


[1] Kullback S, Leibler RA. On information and sufficiency. Annals of Mathematical Statistics. 1951;22(1):79–86.

[2] Kullback S. Letter to the Editor: The Kullback-Leibler distance. The American Statistician. 1987;41(4):340–341.

[3] Rényi A. On measures of information and entropy. In: Neyman J, editor. Proceedings of the Fourth Berkeley Symposium on Mathematics, Statistics and Probability. vol. 1. Statistical Laboratory of the University of California: Berkeley, CA: University of California Press; 1961. p. 547–561.

[4] Nielsen F. Limits from l'Hôpital rule: Shannon entropy as limit cases of Rényi and Tsallis entropies. École Polytechnique/Sony Computer Science Laboratorie, Inc; 2010.

[5] Hellinger E. Neue Begründung der Theorie quadratischer Formen von unendlichvielen Veränderlichen. Journal für die reine und angewandte Mathematik. 1909;136:210–271.

[6] Pardo L. Statistical Inference Based on Divergence Measures. Boca Raton, FL: Chapman & Hall/CRC; 2006.
